# A Serological Survey on Swine Brucellosis Using Standard Procedures, Dot Blot, and Western Blot in Finisher Pigs in Central-North Italy

**DOI:** 10.3390/vetsci5040086

**Published:** 2018-10-03

**Authors:** Fabrizio Bertelloni, Mario Forzan, Barbara Turchi, Simona Sagona, Maurizio Mazzei, Antonio Felicioli, Filippo Fratini, Domenico Cerri

**Affiliations:** Department of Veterinary Science, University of Pisa, Viale delle Piagge 2, 56124 Pisa, Italy; mario.forzan@unipi.it (M.F.); barbara.turchi@unipi.it (B.T.); simonasagona@tiscali.it (S.S.); maurizio.mazzei@unipi.it (M.M.); antonio.felicioli@unipi.it (A.F.); filippo.fratini@unipi.it (F.F.); domenico.cerri@unipi.it (D.C.)

**Keywords:** swine, brucellosis, serology, Dot Blot, Western Blot

## Abstract

In recent years, *Brucella suis* has been sporadically reported in Italy in domestic and wild swine. Since standard serological tests can determine false positive results, the development of alternative tests with improved sensitivity and specificity is rather essential. We analyzed 1212 sera collected at slaughterhouse from healthy pigs belonging to 62 farms of North-Central Italy. Sera were tested by Rose Bengal Test, Complement Fixation Test, and subsequently by a Dot Blot (DB) and Western Blot assays (WB). Only one serum resulted positive to all tests, indicating that swine brucellosis has a very limited spread. DB and WB could represent a support to the available serological tests; however, further studies to validate these tests are needed. In the presence of reemerging diseases, a prompt and continuous monitoring design is necessary to acquire epidemiological information for the subsequent application of specific health emergency plans.

## 1. Introduction

Swine brucellosis is primarily caused by *Brucella suis* (*B. suis*) [1]. *B. suis* can be divided into five biovars of which 1, 2, and 3 are the most relevant for pigs and are globally distributed [2,3]. Brucellosis caused by *Brucella suis* biovar 2 (*B. suis* bv. 2) is emerging in Europe, and although this biovar is not pathogenic for humans, it could be a cause of reproductive failure in pigs, which results in important economic losses for the swine industry [2,4,5,6,7].

Wild boars and hares represent reservoir hosts for *B. suis* bv. 2; these animals are the main sources of infection for domestic pigs, contributing to the spreading of the disease [2,8]. Moreover, wild boars and hares imported for hunting purposes could represent sources of introduction of the pathogen in infection-free areas [8,9]. Recently, *B. suis* bv. 2 was reported for the first time in wild boars [9,10] and later it was reported in swine in different Italian regions [11,12].

Serology plays an important role in brucellosis surveillance and eradication [13]. Nowadays, according to World Organization for Animal Health (OIE) [14], several validated serological tests are available for brucellosis diagnosis in swine: Rose Bengal Test (RBT), Fluorescence Polarisation Assay (FPA), Complement Fixation Test (CFT), and Indirect/Competitive Enzyme-Linked Immunosorbent Assay (I/C ELISA). All these serological tests are routinely used and are based on the detection of antibodies against the smooth lipopolysaccharides (sLPS) of smooth strains. Although these serological tests are globally validated, each of them shows limitations, especially for the screening of individual animals, in particular in swine serum samples [14].

Particularly, it is well documented that antibodies against *Yersinia enterocolitica* O:9 cross-react with *Brucella* sLPS antigens [15]. *Y. enterocolitica* O:9 is particularly widespread in swine populations [16] and represents a frequent cause of false positive serological reactions (FPSR) [13,14]. For these reasons, several authors developed different immunoblotting methods in order to improve sensitivity and specificity of the serological diagnosis [17,18,19].

The aim of this survey was to evaluate the diffusion of *Brucella* spp. in domestic pigs of North-Central Italy by a serological investigation of samples collected at the slaughterhouse from healthy animals employing standard methods and immune assays.

## 2. Materials and Methods

From September to December 2015, 1212 swine blood samples were collected at slaughterhouses from healthy animals belonging to 62 different farms located in five different Italian regions: 33 in Lombardy (660 sera), 15 in Tuscany (274 sera), 7 in Emilia Romagna (138 sera), 5 in Veneto (100 sera), and 2 in Piedmont (40 sera) (Table S1). When it was feasible, from each farm, sera from 20 pigs were collected. Blood samples were quickly transported in refrigerated condition; obtained sera were stored at −20 °C until processed.

All collected sera were screened for anti-brucella antibody by Rose Bengal Test [14]. The sera from farms in which at least one positive sample was present were also analyzed by Complement Fixation Test [14] and by Dot Blot assay (DB). Antigens prepared from a smooth strain of *B. abortus* W99 were produced by “Istituto Zooprofilattico della Lombardia e dell’Emilia Romagna Bruno Ubertini, Brescia” and by “Istituto Zooprofilattico Sperimentale dell’Abruzzo e del Molise G. Caporale, Teramo” for CFTs and for RBTs, respectively.

Antigen employed in DB was Brucellergene OCB (Rhône-Mérieux, Lyon, France), produced from *Brucella melitensis* rough strain B115. DB was performed according to Iovinella et al. [20], with modifications. The antigen (2 µL) was adsorbed on a 0.45 µm size nitrocellulose membrane (Thermo Fisher Scientific, Waltham, MA, USA) and incubated overnight in 3% semi-skimmed milk, 0.05% Tween 20, 100 mM phosphate buffer saline, pH 7.5. The membrane was exposed to serum samples at different concentrations (1:50, 1:100, 1:200), with different incubation times (15, 30, and 45 min) and with or without heat treatment at 58 °C ± 2 °C for 60 min. Afterwards, the membrane was incubated for 1 h at Room Temperature (RT) with a horseradish peroxidase (HRP)-conjugated polyclonal Rabbit anti-Pig IgG-(H+L) antibody (Bethyl Laboratories, Montgomery, TX, USA) diluted 1:10000. The reaction was detected by Immun-Star™ WesternC™ Kit (Biorad Laboratories, Richmond, CA, USA) using a Nikon D5100 camera [21]. Sera scored positive by DB were subsequently analyzed by Western Blot assay (WB) according to Iovinella et al. [20]. The Brucellergene total protein content was measured by Qubit 2.0 Fluorometer (Invitrogen, Waltham, MA, USA). Ten micrograms of Brucellergene total protein were loaded into 12% T and 7.5% T, 2.6% C separating polyacrylamide gels (1.5 mm thick); a 10–250 kDa pre-stained protein Sharpmass™ V plus protein MW marker (Euroclone, Milan, Italy) was used. Sodium Dodecyl Sulphate - PolyAcrylamide Gel Electrophoresis (SDS-PAGE) was performed at 20 mA/ gel at 15 °C using SE 260 mini vertical electrophoresis (GE Healthcare, Little Chalfont Buckinghamshire, UK). Gels were stained in Coomassie brilliant G colloidal solution, scanned by an Epson Perfection V750 Pro, and elaborated by Image J software (National Institutes of Health, Maryland, USA) [22]. Proteins were transferred on the nitrocellulose membrane by ECL TE 70 PWR Semi-dry transfer unit (GE Healthcare) at 75 mA for 4 h and 30 min. The membrane was processed as described for DB; serum concentration, incubation time and heat treatment were chosen on the basis of DB results (1:200, 30 min, and heat treatment at 58 °C ± 2 °C for 60 min, respectively). In order to avoid false positive serological reactions (FPSRs) due to *Y. enterocolitica* O:9, a hyperimmune anti-Yersinia rabbit serum was also tested both by DB and by WB using Goat anti-Rabbit IgG (H+L)-HRP conjugate (Biorad laboratories) as secondary antibody.

All employed antigens were provided by “Istituto Zooprofilattico Sperimentale dell’Abruzzo e del Molise G. Caporale, Teramo”.

## 3. Results

Among 1212 serum samples, only one serum resulted positive to RBT and CFT (titer 1:4 corresponding to 20 International Complement Fixation Test Units for milliliter—ICFTU/mL) and was confirmed by Dot Blot assay. A second serum that was negative to RBT resulted positive to CFT (titer 1:4 corresponding to 20 ICFTU), but it scored negative when tested by Dot Blot. Both these sera obviously belonged to the same farm, located in South Tuscany (Siena province). The best DB experimental conditions were: serum dilution 1:200 at 30 min of incubation (Figure S1, Table S2). Heat treatment of sera was useful for the interpretation of the negative samples. The positive serum was also confirmed by WB (Figures S2 and S3); this test highlighted a band of 53.5 kDa corresponding to the one with the same molecular weight and the same relative mobility (Rm) observed by SDS-PAGE. Hyperimmune anti-Yersinia rabbit serum resulted negative in both assays (Figure S4, Table S3).

## 4. Discussion

*Brucella suis* biovar 2 has been recently introduced in Italy [9,10,11,12]. This bacterium is generally considered a pathogen only for swine that represent, along with wild boars and hares, the most important host for this biovar [2]. However, human infection caused by *B. suis* biovar 2 has been described in France in immuno-compromised hunters [23]. Furthermore, rare cases of asymptomatic infection have been reported in Europe in ruminants after exposure to infected wild boars [24,25]. For these reasons, a constant serological monitoring of wild and domestic swine populations could be essential in order to acquire as much epidemiological information as possible.

In Italy, pig farms are mainly located in the central and northern parts of the country [26]; consequently, the improvement of swine health monitoring in this area is advisable. To the best of our knowledge, there is only one report on the seroprevalence of *Brucella* spp. in breeding pigs in Central-North Italy [11].

Our survey aimed to increase the epidemiological information concerning swine brucellosis in this geographical area. Based on our results, swine brucellosis seems to have a very limited spread. In fact, only one sample resulted positive. One serum resulted positive to CFT but not to RBT and DB. CFT is more specific than RBT, but it is not capable of eliminating the FPSR problem, and can be recommended only as a complementary test, especially in swine, as suggested by WHO [14]. To consolidate the obtained serological results and to exclude possible cross-reactions with other bacteria, two different kinds of antigens were employed: one based on LPS (RBT and CFT) and the other one of protein nature (DB and WB). In our study, therefore, only the serum that resulted positive to all tests employed was considered as positive.

The positive serum came from a free ranged farm of “cinta senese” pigs, in South Tuscany (Siena province), a region characterized by the presence of many wild boars [27] and close to the area where *B. suis* bv. 2 was reported in pigs for the first time [11]. A retrospective investigation highlighted that the positive herd was a free ranged farm of “cinta senese” pigs, where animals could frequently come into contact with wild boars.

Although serological tests cannot discriminate among *Brucella* species, it seems plausible to assume that *B. suis* bv. 2 could be involved in this case, considering also that Tuscany is free from bovine and ovine brucellosis.

Brucellergene was previously employed in swine for in vitro serological test, such as ELISA [13], and for in vivo skin test [7], showing significant performance, especially in term of specificity and in the ability to distinguish FPSR. Moreover, Brucellergene is safe to handle, standardized, and, in some countries, commercially available. For these reasons, it was chosen as an antigen for confirmatory tests. Our results confirm its validity and ease of use in swine brucellosis serological diagnosis.

WB assay resulted useful to confirm and support traditional serology, as also reported previously by other authors [17,18,19]. However, WB execution requires specialized and trained personnel, it is time consuming, and previous work has showed that its sensitivity is low [28]. In this perspective, DB, although its sensibility is probably low, is less time consuming, easier to perform, and applicable to a higher number of samples in comparison to WB. In our study, WB confirmed the results obtained by DB. However, before this method could be applied to support conventional serological tests, such as RBT and CFT, it would be necessary to perform more accurate investigations to precisely evaluate and define its specificity and sensitivity.

## 5. Conclusions

Despite the recent reports, swine brucellosis seems to have a limited diffusion in the investigated area. However, considering the possible hazard for farmed animals, especially swine, and the possible risk for humans, a continuous monitoring plan is advisable. In light of the considerable number of animals to test, slaughterhouses represent the best places for sampling. Serology is a useful and valid tool to obtain epidemiological data, but the development of methods with increased performance characteristics is required. DB and WB could represent possible candidates for this purpose, but, on the other hand, it is necessary to perform additional studies to fully validate those experimental approaches in order to evaluate their performances and usefulness.

## Supplementary Materials

The following are available online at http://www.mdpi.com/2306-7381/5/4/86/s1, Table S1: Farms included in this study; Figure S1: SDS-PAGE of Brucellergene antigen; Figure S2: Dot Blot at different experimental condition: a = serum without heat treatment; b = serum after heat treatment; 1:100 and 1:200 = sera dilution in PBS; 45′, 30′, and 15′ = sera incubation times in minutes; + serum and − serum = positive and negative sera, respectively; Table S2: Optical density measured from Dot Blot assay from different experimental conditions; Figure S3: Dot Blot of different sera: P+ = positive serum; P+/− = serum positive to CFT and negative to RBT; P− = negative serum; R Yersinia + = rabbit serum positive to *Yersinia enterocolitica* O:9; R Yersinia − = SPF rabbit serum; Table S3: Optical density measured from Dot Blot assay of different serum samples; Figure S4: Western Blot analysis: proteins of Brucellergene were separated by SDS-PAGE and blotted on nitrocellulose membrane using a semidry system. +: positive serum; −: negative serum.

## References

[1] Olsen S.C., Garin-Bastuji B., Blasco J.M., Nicola A.M., Samartino L., Zimmerman J.J., Karriker L.A., Ramirez A., Schwartz G.W. (2012). Brucellosis. Stevenson, Diseases of Swine.

[2] Godfroid J., Scholz H.C., Barbier T., Nicolas C., Wattiau P., Fretin D., Whatmore A.M., Cloeckaert A., Blasco J.M., Moriyon I. (2011). Brucellosis at the animal/ecosystem/human interface at the beginning of the 21st century. Prev. Vet. Med..

[3] Duvnjak S., Račić I., Špičić S., Zdelar-Tuk M., Reil I., Cvetnić Ž. (2015). Characterisation of *Brucella suis* isolates from Southeast Europe by multi-locus variable-number tandem repeat analysis. Vet. Microbiol..

[4] Cvetnić Z., Spicić S., Toncić J., Majnarić D., Benić M., Albert D., Thiébaud M., Garin-Bastuji B. (2009). *Brucella suis* infection in domestic pigs and wild boar in Croatia. Rev. Sci. Tech..

[5] Ferreira A.C., Almendra C., Cardoso R., Pereira M.S., Beja-Pereira A., Luikart G., de Sá M.I.C. (2012). Development and evaluation of a selective medium for *Brucella suis*. Res. Vet. Sci..

[6] Szulowski K., Iwaniak W., Weiner M., Zlotnicka J. (2013). Characteristics of *Brucella* strains isolated from animals in Poland. Pol. J. Vet. Sci..

[7] Dieste-Pérez L., Blasco J.M., De Miguel M.J., Marín C.M., Barberán M., Conde-Álvarez R., Moriyón I., Muñoz P.M. (2014). Performance of skin tests with allergens from *B. melitensis* B115 and rough *B. abortus* mutants for diagnosing swine brucellosis. Vet. Microbiol..

[8] Kreizinger Z., Foster J.T., Rónai Z., Sulyok K.M., Wehmann E., Jánosi S., Gyuranecz M. (2014). Genetic relatedness of *Brucella suis* biovar 2 isolates from hares, wild boars and domestic pigs. Vet. Microbiol..

[9] De Massis F., di Provvido A., di Sabatino D., di Francesco D., Zilli K., Ancora M., Tittarelli M. (2012). Isolation of *Brucella suis* biovar 2 from a wild boar in the Abruzzo Region of Italy. Vet. Ital..

[10] Bergagna S., Zoppi S., Ferroglio E., Gobetto M., Dondo A., Di Giannatale E., Gennero M.S., Grattarola C. (2009). Epidemiologic survey for *Brucella suis* biovar 2 in a wild boar (*Sus scrofa*) population in northwest Italy. J. Wildl. Dis..

[11] Barlozzari G., Franco A., Macrì G., Lorenzetti S., Maggiori F., Dottarelli S., Maurelli M., Di Giannatale E., Tittarelli M., Battisti A., Gamberale F. (2015). First report of *Brucella suis* biovar 2 in a semi free-range pig farm, Italy. Vet. Ital..

[12] Pilo C., Tedde M.T., Orrù G., Addis G., Liciardi M. (2015). *Brucella suis* infection in domestic pigs in Sardinia (Italy). Epidemiol. Infect..

[13] McGiven J.A., Nicola A., Commander N.J., Duncombe L., Taylor A.V., Villari S., Dainty A., Thirlwall R., Bouzelmat N., Perrett L.L. (2012). An evaluation of the capability of existing and novel serodiagnostic methods for porcine brucellosis to reduce false positive serological reactions. Vet. Microbiol..

[14] World Organization for Animal Health (2017). Manual of Diagnostic Tests and Vaccines for Terrestrial Animals. Brucella abortus, B. melitensis and B. suis Office International Des Epizooties.

[15] Jungersen G., Sørensen V., Giese S.B., Stack J.A., Riber U. (2006). Differentiation between serological responses to *Brucella suis* and *Yersinia enterocolitica* serotype O:9 after natural or experimental infection in pigs. Epidemiol. Infect..

[16] EFSA (European Food Safety Authority) and ECDC (European Centre for Disease Prevention and Control) (2016). The European Union summary report on trends and sources of zoonoses, zoonotic agents and food-borne outbreaks in 2015. EFSA J..

[17] Spencer S.A., Broughton E.S., Hamid S., Young D.B. (1994). Immunoblot studies in the differential diagnosis of porcine brucellosis: An immunodominant 62 kDa protein is related to the mycobacterial 65 kDa heat shock protein (HSP-65). Vet. Microbiol..

[18] Corrente M., Desario C., Greco G., Buonavoglia D., Pratelli A., Madio A., Scaltrito D., Consenti B., Buonavoglia C. (2004). Development of a western blotting assay to discriminate *Brucella* spp. and *Yersinia enterocolitica* O:9 infections in sheep. New Microbiol..

[19] Wareth G., Melzer F., Weise C., Neubauer H., Roesler U., Murugaiyan J. (2015). Proteomics-based identification of immunodominant proteins of Brucellae using sera from infected hosts points towards enhanced pathogen survival during the infection. Biochem. Biophys. Res. Commun..

[20] Iovinella I., Dani F.R., Niccolini A., Sagona S., Michelucci E., Gazzano A., Turillazzi S., Felicioli A., Pelosi P. (2011). Differential expression of odorant-binding proteins in the mandibular glands of the honey bee according to caste and age. J. Proteome Res..

[21] Khoury M.K., Parker I., Aswad D.W. (2010). Acquisition of chemiluminescent signals from immunoblots with a digital single-lens reflex camera. Anal. Biochem..

[22] Abramoff M.D., Magalhaes P.J., Ram S.J. (2004). Image Processing with Image. J. Biophoton. Int..

[23] Pappas G. (2010). The changing *Brucella* ecology: Novel reservoirs, new threats. Int. J. Antimicrob. Agents.

[24] Fretin D., Mori M., Czaplicki G., Quinet C., Maquet B., Godfroid J., Saegerman C. (2013). Unexpected *Brucella suis* biovar 2 infection in a dairy cow, Belgium. Emerg. Infect. Dis..

[25] Szulowski K., Iwaniak W., Weiner M., Złotnicka J. (2013). *Brucella suis* biovar 2 isolations from cattle in Poland. Ann. Agric. Environ. Med..

[26] ISTAT 6° Censimento Generale dell’Agricoltura. https://www.istat.it/it/archivio/66591.

[27] Carnevali L., Pedrotti L., Riga F., Toso S. (2009). Banca Dati Ungulati: Status, distribuzione, consistenza, gestione e prelievo venatorio delle popolazioni di Ungulati in Italia. Rapporto 2001–2005. Biol. Cons. Fauna.

[28] Kittelberger R., Hilbink F., Hansen M.F., Penrose M., de Lisle G.W., Letesson J.J., Garin-Bastuji B., Searson J., Fossati C.A., Cloeckaert A. (1995). Serological crossreactivity between *Brucella abortus* and *Yersinia enterocolitica* O:9 I immunoblot analysis of the antibody response to *Brucella* protein antigens in bovine brucellosis. Vet. Microbiol..

