# Supplementary material for: A Serological Survey on Swine Brucellosis Using Standard Procedures, Dot Blot, and Western Blot in Finisher Pigs in Central-North Italy
Source: Vet Sci. 2018 Oct 3;5(4):86. doi: 10.3390/vetsci5040086 (PMC6313843; doi:10.3390/vetsci5040086)
Supplement: Supplementary file 1 [file vetsci-05-00086-s001.pdf]

Supplementary Materials: A Serological Survey on Swine Brucellosis Using Standard Procedures, Dot Blot, and Western Blot in Finisher Pigs in Central-North Italy

Fabrizio Bertellonia, Mario Forzana, Barbara Turchi, Simona Sagona, Maurizio Mazzei, Antonio Felicioli, Filippo Fratini and Domenico Cerri

Table S1. Farms included in this study.

Date	Farm Number	Province	Italian Region	Analyzed sera
28/09/2015	7	MO	Emilia Romagna	20
29/09/2015	12	RE	Emilia Romagna	20
30/09/2015	13	MO	Emilia Romagna	18
05/10/2015	21	MO	Emilia Romagna	20
28/10/2015	42	PR	Emilia Romagna	20
23/11/2015	59	PC	Emilia Romagna	20
30/11/2015	61	MO	Emilia Romagna	20
22/09/2015	1	BG	Lombardy	20
22/09/2015	2	PV	Lombardy	20
23/09/2015	4	PV	Lombardy	20
23/09/2015	6	BS	Lombardy	20
28/09/2015	9	CR	Lombardy	20
29/09/2015	11	PV	Lombardy	20
30/09/2015	14	BS	Lombardy	20
30/09/2015	15	PV	Lombardy	20
05/10/2015	19	MN	Lombardy	20
06/10/2015	22	BS	Lombardy	20
06/10/2015	23	BG	Lombardy	20
07/10/2015	25	LO	Lombardy	20
08/10/2015	26	BS	Lombardy	20
12/10/2015	27	CR	Lombardy	20
13/10/2015	28	PV	Lombardy	20
13/10/2015	29	MI	Lombardy	20
14/10/2015	30	PV	Lombardy	20
14/10/2015	31	BG	Lombardy	20
15/10/2015	32	CR	Lombardy	20
20/10/2015	33	CR	Lombardy	20
20/10/2015	34	CR	Lombardy	20
20/10/2015	35	PV	Lombardy	20
21/10/2015	36	BS	Lombardy	20
22/10/2015	39	LO	Lombardy	20
27/10/2015	41	CR	Lombardy	20
02/11/2015	43	CR	Lombardy	20
03/11/2015	45	BS	Lombardy	20
09/11/2015	47	CR	Lombardy	20
11/11/2015	48	CR	Lombardy	20
11/11/2015	49	BS	Lombardy	20
11/11/2015	50	CR	Lombardy	20
18/11/2015	54	BS	Lombardy	20

Vet. Sci. **2018**, *5*, 86; doi:10.3390/vetsci5040086

18/11/2015	55	CR	Lombardy	20
30/11/2015	60	CR	Lombardy	20
05/10/2015	20	CN	Piedmont	20
21/10/2015	37	CN	Piedmont	20
23/09/2015	5	PT	Tuscany	6
28/09/2015	8	AR	Tuscany	20
28/09/2015	10	PT	Tuscany	10
01/10/2015	16	SI	Tuscany	20
01/10/2015	18	FI	Tuscany	20
07/10/2015	24	SI	Tuscany	20
22/10/2015	38	PI	Tuscany	20
27/10/2015	40	AR	Tuscany	20
02/11/2015	44	AR	Tuscany	20
12/11/2015	51	AR	Tuscany	20
17/11/2015	53	GR	Tuscany	20
19/11/2015	56	PI	Tuscany	20
19/11/2015	57	PI	Tuscany	20
30/11/2015	62	SI	Tuscany	18
22/09/2015	3	VI	Veneto	20
01/10/2015	17	VR	Veneto	20
03/11/2015	46	VI	Veneto	20
16/11/2015	52	VR	Veneto	20
23/11/2015	58	RO	Veneto	20
		Total		1212

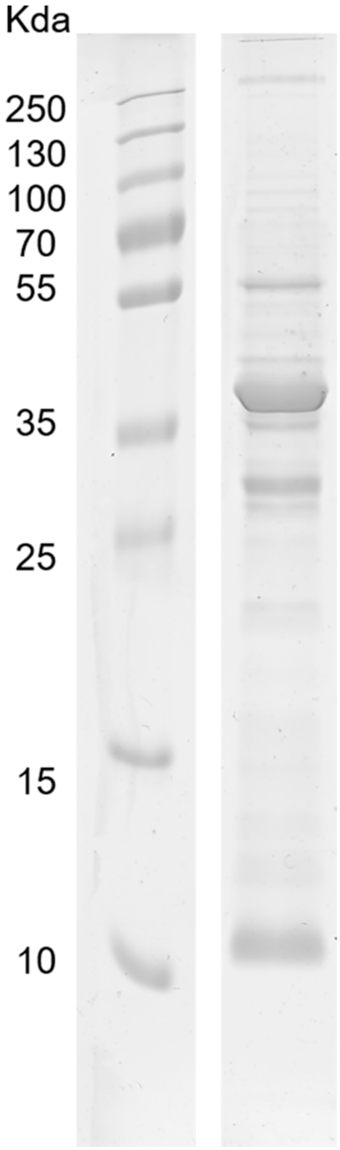

Figure S1. SDS-PAGE of Brucellergene antigen.

Table S2. Optical density measured from Dot Blot assay from different experimental conditions.

Heat Treatment	Sera Dilution	Incubation Time (minutes)	Sera	Area	Mean	Min	Max
WHITOUT	1:100	45	+	2828	169.104	138	184
WHITOUT	1:100	30	+	2828	125.677	120	138
WHITOUT	1:100	15	+	2828	138.257	124	156
AFTER	1:100	45	+	2828	142.386	125	156
AFTER	1:100	30	+	2828	134.916	123	143
AFTER	1:100	15	+	2828	159.807	135	172
WHITOUT	1:200	45	+	2828	142.519	132	152
WHITOUT	1:200	30	+	2828	152.520	129	175
WHITOUT	1:200	15	+	2828	157.149	139	168
AFTER	1:200	45	+	2828	139.536	125	152
AFTER	1:200	30	+	2828	151.633	130	172
AFTER	1:200	15	+	2828	158.683	141	172
WHITOUT	1:100	45	-	2828	113.408	110	118
WHITOUT	1:100	30	-	2828	116.254	112	120

WHITOUT	1:100	15	-	2828	116.603	111	123
AFTER	1:100	45	-	2828	108.964	105	113
AFTER	1:100	30	-	2828	105.758	105	107
AFTER	1:100	15	-	2828	125.274	120	129
WHITOUT	1:200	45	-	2828	109.525	106	154
WHITOUT	1:200	30	-	2828	105.358	104	107
WHITOUT	1:200	15	-	2828	112.778	109	119
AFTER	1:200	45	-	2828	107.990	105	113
AFTER	1:200	30	-	2828	107.048	105	109
AFTER	1:200	15	-	2828	112.307	107	117

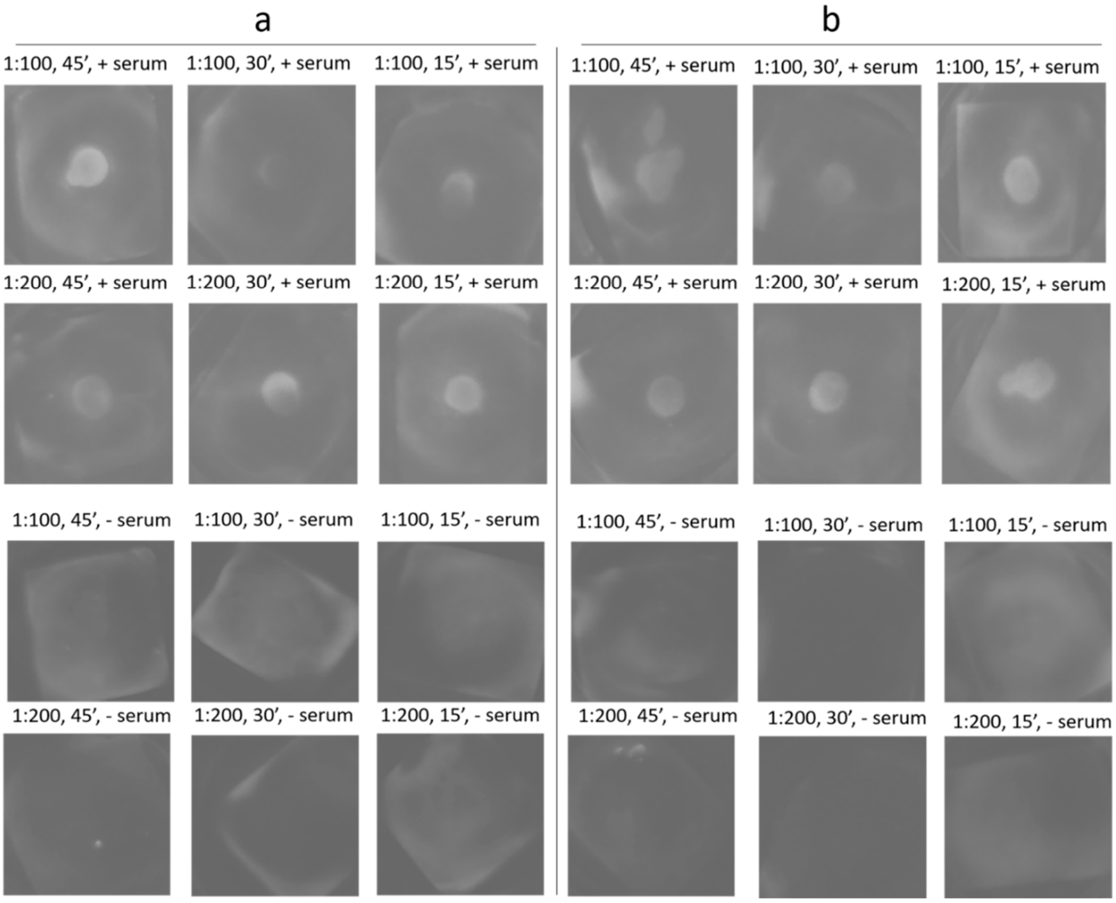

Figure S2. Dot Blot at different experimental condition: a = serum without heat treatment; b = serum after heat treatment; 1:100 and 1:200 = sera dilution in PBS; 45′, 30′, and 15′ = sera incubation times in minutes; + serum and – serum = positive and negative sera, respectively.

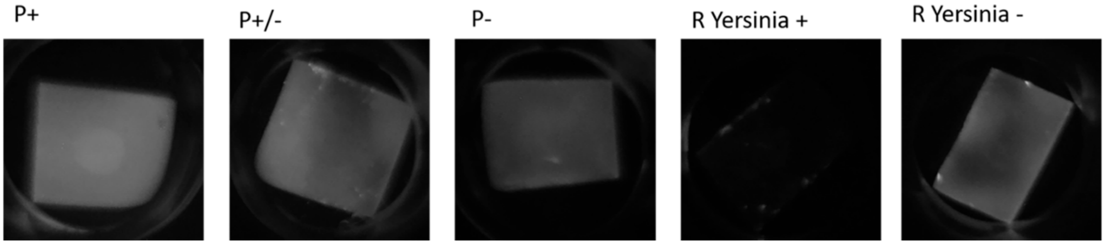

Figure S3. Dot Blot of different sera: P+ = positive serum; P+/− = serum positive to CFT and negative to RBT; P− = negative serum; R Yersinia + = rabbit serum positive to Yersinia enterocolitica O:9; R Yersinia − = SPF rabbit serum.

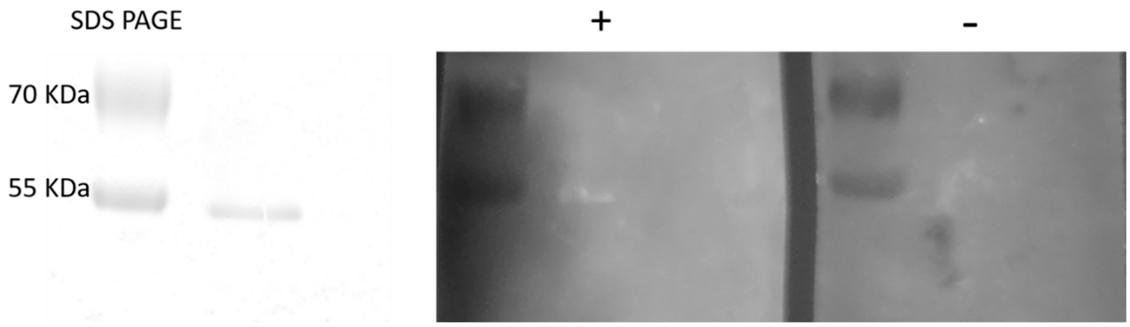

Figure S4. Western Blot analysis: proteins of Brucellergene were separated by SDS-PAGE and blotted on nitrocellulose membrane using a semidry system. +: positive serum; −: negative serum.

Table S3. Optical density measured from Dot Blot assay of different serum samples.

Title	Area	Mean	Min	Max
P+	2828	117.138	108	125
P+/-	2828	66.787	50	85
P-	2828	62.552	54	71
R Yersinia +	2828	14.496	9	21
R Yersinia −	2828	53.718	45	69

Dot Blot of different sera: P+ = positive serum; P+/− = serum positive to CFT and negative to RBT; P− = negative serum; R Yersinia + = rabbit serum positive to *Yersinia enterocolitica* O:9; R Yersinia − = SPF rabbit serum.

© 2018 by the authors. Licensee MDPI, Basel, Switzerland. This article is an open access article distributed under the terms and conditions of the Creative Commons Attribution (CC BY) license (http://creativecommons.org/licenses/by/4.0/).
